# People can understand descriptions of motion without activating visual motion brain regions

**DOI:** 10.3389/fpsyg.2013.00537

**Published:** 2013-08-28

**Authors:** Swethasri Dravida, Rebecca Saxe, Marina Bedny

**Affiliations:** Saxe Lab for Social Cognitive Neuroscience, Department of Brain and Cognitive Sciences, Massachusetts Institute of TechnologyCambridge, MA, USA

**Keywords:** language, motion, embodiment, simulation, MT/MST, right superior temporal sulcus, inferior parietal lobule, superior parietal lobule

## Abstract

What is the relationship between our perceptual and linguistic neural representations of the same event? We approached this question by asking whether visual perception of motion and understanding linguistic depictions of motion rely on the same neural architecture. The same group of participants took part in two language tasks and one visual task. In task 1, participants made semantic similarity judgments with high motion (e.g., “to bounce”) and low motion (e.g., “to look”) words. In task 2, participants made plausibility judgments for passages describing movement (“A centaur hurled a spear … ”) or cognitive events (“A gentleman loved cheese …”). Task 3 was a visual motion localizer in which participants viewed animations of point-light walkers, randomly moving dots, and stationary dots changing in luminance. Based on the visual motion localizer we identified classic visual motion areas of the temporal (MT/MST and STS) and parietal cortex (inferior and superior parietal lobules). We find that these visual cortical areas are largely distinct from neural responses to linguistic depictions of motion. Motion words did not activate any part of the visual motion system. Motion passages produced a small response in the right superior parietal lobule, but none of the temporal motion regions. These results suggest that (1) as compared to words, rich language stimuli such as passages are more likely to evoke mental imagery and more likely to affect perceptual circuits and (2) effects of language on the visual system are more likely in secondary perceptual areas as compared to early sensory areas. We conclude that language and visual perception constitute distinct but interacting systems.

## Introduction

What is the relationship between sensory perception and concepts? Cognitive neuroscience offers one approach to this question: We can ask whether sensory perception and language depend on the same neural machinery. Does understanding the sentence “The man jumped out of the car” depend on the same neural circuits as visually perceiving men, cars, and jumping? Words and sentences that describe motion offer a particularly good opportunity to test this hypothesis, because of the long history of studying the neural representation of visually perceived motion. In the current study, we examine possible links between language and perception by comparing the neural mechanisms underlying comprehension of language describing motion and actual visual perception of motion.

Visual perception of motion is supported by a network of temporal and parietal brain regions. The earliest cortical area that selectively responds to motion is the middle temporal complex (MT/MST) of posterior lateral temporal cortex (Tootell et al., 1995; Born and Bradley, 2005). Individual neurons in MT/MST are tuned to specific directions and speeds of motion (Dubner and Zeki, 1971). Damage to MT/MST in humans results in akinotopsia, a selective deficits in motion vision. Severely akinotopsic patients fail to perceive moving objects as traversing smoothly through space; for example an approaching car appears to jump from a far away location to close up (Zeki, 1991). By contrast, the same patients have near normal color and form vision and have no apparent deficits in auditory or tactile motion perception. In sum, MT/MST is a motion selective region driven primarily by inputs from the visual modality.

In addition to MT/MST, higher-order areas in the temporal and parietal lobes also contribute to visual motion perception. While MT/MST responds to both random and coherent motion of any kind, a right-lateralized region along the superior temporal suclus (RSTS) is involved in perceiving only certain kind of motion: namely, biological human and animal motion (Grossman et al., 2000; Grossman and Blake, 2002; Saygin, 2007; Grosbras et al., 2012; Gilaie-Dotan et al., 2013). Transiently disrupting RSTS activity, using TMS, selectively impairs visual perception of biological but not similar non-biological motion (Grossman et al., 2005). Several regions in the parietal lobe, including the intra parietal sulcus (IPS) and right inferior parietal lobule (IPL) also contribute to higher-order aspects of motion perception. Unlike the selective response to *visual* motion in MT/MST, these parietal regions respond to visual, tactile, and auditory motion alike (Griffiths et al., 1998; Lewis et al., 2000; Bremmer et al., 2001). The parietal cortex contributes to the perception of complex and ambiguous motion, including apparent motion (Battelli et al., 2003). Responses to motion in the parietal cortex are closely related to an animal's subjective percept, rather then to the physical properties of the visual stimulus. For example, parietal neurons respond to the likely future direction of motion (Williams et al., 2003).

In this paper we leverage existing knowledge of the visual motion system to gain insight into the link between sensory perception and language. We ask: what is the role of these visual motion regions in comprehension of language that describes motion? We can distinguish between three hypotheses about the relationship of language and perception that predict different patterns of results. First, understanding concrete language could depend on simulation of modality-specific experiences. If so, comprehension of a phrase such as “the man jumped onto the windowsill” should require activation in all of the regions that would be recruited while watching a man jumping, including MT/MST, STS, IPS, and IPL. Perception should permeate all aspects of language processing, including the retrieval of individual word meanings.

Second, a more limited hypothesis we will call optional interactivity, is that linguistic depictions of events optionally recruit some areas in common with sensory perception. For example, perceptual neural representations might be activated as a result of spontaneous imagery during language comprehension. On this view, visual motion areas might be more likely to respond to linguistic descriptions that elicit such imagery e.g., passages but not single words. These responses would occur via top-down influence of linguistic neural representations on visual motion circuits, and should therefore be more likely in higher-order rather than early perceptual areas. Specifically, parietal multi-modal motion neural representations might be evoked by linguistic stimuli, while early modality-specific neural representations in regions like MT/MST might require direct visual perception.

Finally, a third hypothesis is that comprehension of linguistic descriptions of motion never recruits perceptual neural representations. This could be due to the modularity of the language system, modularity of perceptual systems or both (Fodor, 1983). Activity would occur in perceptual regions only when participants are viewing or intentionally imaging actual visual motion.

A number of prior studies asked whether brain areas that respond to visual movement also respond to language that describes motion. Initial investigations along these lines appeared to support a strong link between vision and language. Several neuroimaging studies observed responses near MT/MST to action verbs (e.g., to jump) as compared to names of object or animals (Martin et al., 1995; Damasio et al., 2001). These data were taken as evidence that the meanings of action verbs are represented in part as visual motion schema. Understanding a word such as “to jump” obligatorily involves retrieving past visual experiences of seeing jumping.

Subsequent experiments showed, however, that lateral temporal responses to action verbs lie anterior and superior to visual motion responses in MT/MST, in the posterior aspect of the left middle temporal gyrus (pLMTG; Kable et al., 2002, 2005; Bedny et al., 2008; see also Wallentin et al., 2011 for similar findings in the context of a story comprehension task). The functional profile of the action-verb-responsive area is distinct from the motion-selective profile of its perceptual neighbors. The pLMTG responds not only to motion verbs such as “to run” but also to verbs such as “to think” which lack any motion information. Nor is the development of pLMTG dependent on visual motion experience. Individuals who have never seen jumping or running due to congenital blindness, show normal responses to action verbs in the pLMTG (Noppeney et al., 2003; Bedny et al., 2008). These findings suggest that pLMTG responses to motion verbs are driven by their semantic or linguistic properties, rather than by their motion associations. In sum, there is little evidence that MT/MST neural representations of visual motion are evoked automatically during comprehension of action verbs.

On the other hand, it remains possible that multi-modal parietal regions can be recruited both by actual visual motion and by words that describe motion, as suggested by the optional interactivity hypothesis. Several studies have reported larger responses to motion words than non-motion words in parietal regions (Noppeney et al., 2005; Mahon et al., 2007; Pobric et al., 2010; Van Dam et al., 2010). For example, a parietal region responded more to action verbs that describe an action of the body (“to wipe,” “to wave”) than abstract verbs (“to appreciate,” “to judge”; Van Dam et al., 2010). However, no study has yet investigated whether the parietal regions recruited by motion words are the *same* parietal regions that respond to visual motion. The parietal cortex also contains regions that are responsive specifically to linguistic information, and regions that are sensitive to abstract properties of actions (Catani and Jones, 2005; Fogassi et al., 2005). It is possible that in parietal cortex, as in temporal cortex, visual motion and linguistic responses occur in neighboring but distinct patches.

Another key open question is whether richer or more vivid linguistic descriptions of motion are more likely to evoke responses in perceptual circuits. Some recent studies have found responses to sentences and passages describing motion events in MT/MST and the right STS (Tettamanti et al., 2005; Saygin et al., 2009; Deen and McCarthy, 2010; McCullough et al., 2012). These data raise the possibly that even early visual motion areas respond to rich motion language such as sentences and passages, but not to single words with motion features. As compared to single words, passages are better stimuli for eliciting spontaneous imagery. Unlike words, which refer to general categories, passages can describe specific instances of motion. For example, the word “to roll” refers to a class of rolling motion. A ball rolling down the street and a pig rolling in the mud describe two visually different events. If passages but not words elicit responses in visual motion areas, this could provide insights into the cognitive role of perceptual responses to language. However, no prior study has directly compared responses to passages and words in visual motion areas, leaving open the possibility that conflicting findings from prior literature are due to other methodological factors.

The goals of the present study were to ask (1) whether motion language is more likely to elicit responses in secondary than early sensory areas and (2) whether passages are more likely to activate perceptual motion areas than single words. We asked the same group of participants to perform three tasks. In task 1, participants read passages consisting of four sentences. Half of the passages contained action verbs and were high in visual motion features, whereas the other half contained cognitive verbs and were low in visual motion features. To ensure attention to text content, participants made semantic plausibility judgments for each sentence in a passage. In task 2, participants made semantic similarity judgments about words that were either high (e.g., to roll) or low (e.g., to smell) in visual motion features. Subsequent to the language tasks, participants saw a visual motion localizer (task 3) with three conditions: biological motion, random motion and luminance change. We localized lateral temporal (MT/MST and STS) and parietal (IPS and IPL) motion areas in each individual participant and asked whether these regions showed responses to either high motion words or high motion passages.

## Materials and methods

### Participants

Eighteen adults (seven males) took part in three experimental tasks during a single fMRI session: motion word-comprehension, motion sentence-comprehension, and visual motion perception. Data from the passage comprehension task were excluded for one participant because they were discovered to have participated in a behavioral pilot study with the same stimuli. All participants were native English speakers and the average age of participants was 23 years (*SD* = 3.3, range 18–30). Participants had no known psychiatric or neurological disabilities and were not currently taking any psychoactive medications. All participants gave informed consent prior to taking part in the study and were compensated $30 per hour.

### Visual motion survey procedure

Words and passages were rated by a separate group of participants on the degree to which they elicit visual motion imagery (scale of 1–7, 7 being high in visual motion; See Appendix for Instructions). The instructions for both surveys were identical except for the examples used (words vs. passages). Participants rated whole passages from task 1 and single words from task 2. Motion ratings were collected through Amazon Mechanical Turk, an online survey system. Participants were screened to be native English speakers through self-report. All participants completed a demographic survey and were asked what language they learned first. If they answered anything other than English, their data were dropped from further analyses. Two separate groups of participants rated the passages (*n* = 73, 34 females, mean age = 31 years, *SD* = 9.8, range 18–61) and words (*n* = 22, 12 females, mean age = 28 years, *SD* = 11.2, range 18–60). Participants were paid $1.50 for rating the passages and $0.25 for rating the words.

### Stimuli

#### Passage stimuli

**Figure 1:**
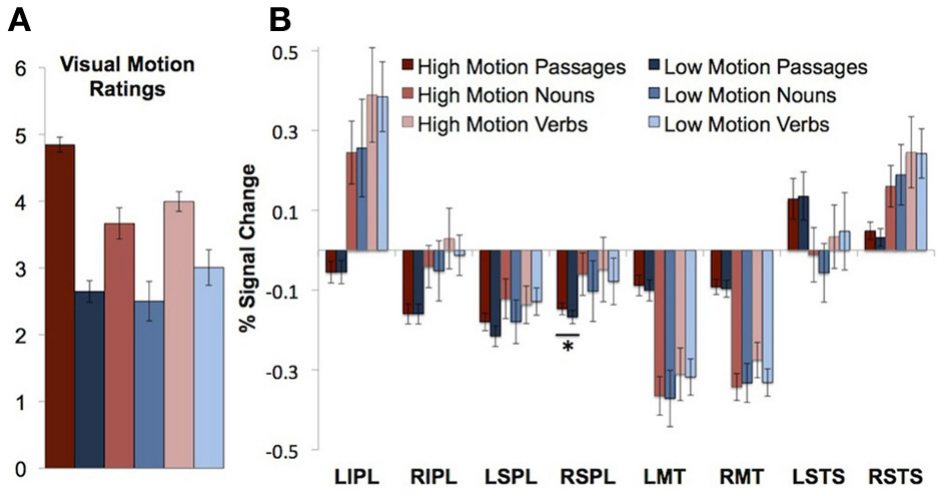
**Visual motion ratings and neural responses to motion passages and words in visual motion ROIs. (A)** Visual motion ratings **(B)** Percentage signal change in visual motion regions of interest during passage and word comprehension. Error bars throughout figure represent standard error of the mean. Asterisks indicate a significant difference between the high and low motion conditions (*P* < 0.05).

Passages consisted of four short sentences each. Sentences were in active voice and consisted of a subject (singular noun), transitive verb followed by an object. Half of the passages contained high motion verbs (“A juror kicked a stool … ”), and half contained low motion cognitive verbs (“An accountant described a painting … ”). The high motion passages were rated as bringing to mind more visual motion than the low motion passages [*t*_(72)_ = 12.71, *P* < 0.0001; See Table 1B for example of passage stimuli]. Average visual motion survey ratings for high and low motion passages are presented in Figure 1A.

Table 1**List of stimuli**.

**Table:** 

**(A) ALL WORD STIMULI**							
**HIGH MOTION NOUNS**							
the aardvark	2.86	the coyote	4.27	the incident	3.14	the prom	4.59
the accident	4.18	the crane	3.23	the lemur	3.59	the quest	4.23
the adventure	4.45	the cyclone	5.59	the lesson	2.50	the reunion	3.32
the alligator	3.32	the dinner	3.18	the lizard	3.36	the rhinoceros	3.45
the alpaca	3.18	the drought	2.50	the llama	3.68	the robbery	4.41
the antelope	4.05	the elephant	3.77	the luncheon	2.81	the rodeo	3.91
the armadillo	2.86	the episode	3.09	the marathon	5.23	the salamander	3.27
the avalanche	5.27	the exam	3.36	the meerkat	3.41	the seminar	2.95
the banquet	3.41	the excursion	3.82	the mongoose	3.36	the sermon	2.41
the beaver	3.50	the falcon	3.86	the mosquito	4.23	the session	2.50
the blizzard	4.32	the famine	2.91	the movie	3.91	the shark	4.73
the brunch	2.95	the festival	4.45	the muskrat	3.73	the shindig	3.27
the burglary	4.00	the funeral	2.68	the nightmare	3.27	the speech	3.59
the butterfly	3.91	the gala	3.45	the octopus	4.00	the spider	3.91
the camel	3.32	the gazelle	4.91	the orangutan	4.05	the storm	4.76
the carnival	4.14	the giraffe	4.09	the ostrich	3.95	the supper	2.95
the caterpillar	2.86	the gopher	3.05	the pageant	3.52	the surgery	3.82
the ceremony	3.95	the gorilla	4.14	the parakeet	3.14	the tornado	5.82
the chameleon	3.27	the hamster	3.73	the peacock	3.68	the tournament	4.50
the chinchilla	2.73	the hedgehog	3.23	the pelican	3.73	the trial	3.23
the cockroach	3.59	the heron	3.27	the pigeon	3.86	the vacation	4.00
the concert	4.18	the holiday	3.36	the platypus	3.09	the vulture	4.41
the contest	3.00	the hurricane	5.45	the porcupine	3.41	the warthog	2.95
the coronation	2.73	the hyena	4.14	the porpoise	3.55	the wedding	3.91
the cougar	3.86	the iguana	2.91	the prank	3.32	the whale	3.95
**LOW MOTION NOUNS**							
the acorn	2.55	the chrysanthemum	2.59	the maple	2.32	the shrub	2.27
the almond	2.23	the clementine	2.50	the mushroom	2.45	the soybean	2.32
the apricot	2.91	the clover	2.86	the oak	3.00	the sycamore	2.57
the artichoke	2.73	the coconut	2.77	the orange	2.55	the tangerine	2.36
the asparagus	2.32	the daffodil	2.68	the papaya	2.50	the turnip	2.36
the birch	2.27	the dandelion	2.55	the parsnip	2.18	the twig	2.32
the branch	3.23	the date	3.59	the pistachio	2.00	the vine	2.50
the bush	2.77	the evergreen	2.68	the pomegranate	2.27	the watercress	2.45
the cactus	2.32	the fern	2.41	the radish	2.05	the weed	2.45
the cantaloupe	2.55	the gourd	2.14	the rhubarb	2.45	the yam	2.23
the carnation	2.77	the grape	2.73	the root	2.36	the zucchini	2.45
the cashew	2.23	the herb	2.23	the rutabaga	2.09		
the cedar	2.50	the kiwi	3.09	the seed	2.41		
**HIGH MOTION VERBS**							
to bounce	4.82	to leap	4.36	to scoot	3.45	to swing	4.64
to climb	4.32	to limp	3.23	to scurry	3.82	to trek	4.18
to crawl	3.36	to meander	2.68	to skip	4.91	to trot	4.00
to dance	5.18	to paddle	4.27	to slide	4.55	to twirl	4.24
to drift	3.00	to prance	3.86	to slither	3.55	to twist	4.36
to drop	4.00	to prowl	3.41	to sneak	3.36	to waddle	3.18
to float	3.14	to revolve	4.68	to spin	4.77	to wade	3.18
to frolic	3.55	to ride	4.95	to stagger	3.27	to walk	4.27
to gallop	4.95	to roam	3.55	to stomp	3.73	to wander	3.82
to glide	4.41	to roll	4.55	to stroll	3.18	to whirl	4.24
to hike	4.41	to rotate	4.86	to strut	3.50	to zigzag	4.55
to hobble	3.33	to saunter	2.67	to stumble	3.68		
to jump	5.27	to scamper	3.55	to swim	4.86		
**LOW MOTION VERBS**							
to admire	3.09	to flare	2.77	to moan	2.55	to sense	2.82
to ascertain	3.05	to flash	3.59	to mumble	2.55	to shimmer	3.27
to babble	2.81	to flicker	2.73	to notice	2.68	to shine	3.05
to behold	3.00	to frisk	3.09	to observe	3.00	to shriek	3.05
to bellow	3.48	to gape	3.05	to ogle	2.32	to sing	3.55
to blare	2.86	to gawk	2.82	to overhear	2.64	to smell	2.77
to blaze	3.27	to gaze	2.27	to overlook	2.91	to sniff	3.09
to buzz	3.41	to glance	2.86	to peek	3.32	to snoop	3.05
to caress	3.55	to gleam	2.41	to peep	2.91	to sparkle	3.41
to chant	2.45	to glimpse	2.91	to peer	3.19	to spy	3.55
to chatter	3.14	to glisten	2.64	to perceive	2.55	to squawk	2.91
to chime	2.68	to glow	3.05	to peruse	2.86	to squeak	3.05
to chuckle	2.82	to groan	2.59	to pet	3.50	to squeal	2.77
to clang	3.23	to growl	2.82	to probe	3.32	to stare	2.95
to click	3.09	to grunt	2.64	to prod	3.00	to stink	2.41
to crackle	3.32	to hiss	2.64	to purr	2.71	to stroke	3.68
to creak	2.64	to hoot	3.18	to recognize	2.23	to tap	4.14
to cry	3.45	to howl	3.27	to reek	2.23	to taste	3.18
to detect	3.23	to hum	2.59	to ring	3.55	to thud	2.91
to discern	2.50	to identify	2.82	to roar	3.71	to twinkle	3.00
to discover	3.73	to inspect	2.95	to rub	3.36	to view	2.77
to eavesdrop	2.41	to investigate	3.59	to rumble	3.82	to wail	3.05
to evaluate	2.68	to jingle	3.41	to rustle	2.86	to whine	3.00
to examine	3.14	to leer	2.50	to scan	2.82	to whisper	2.59
to explore	4.86	to lick	3.91	to scrutinize	2.77	to witness	2.38

**Table:** 

**(B) SAMPLE PASSAGE STIMULI**			
**HIGH MOTION PASSAGES**			
A physicist assassinated his nephew	5.59	A lunatic juggled oranges	5.41
Then a client lifted a prostitute		Then a clown squished tires	
Then a stewardess stabbed a pilot		Then an elephant bumped a barrel	
Then a waitress whacked a bachelor		Then a monkey twirled a baton	
**LOW MOTION PASSAGES**			
A cameraman detested a slave	2.13	A freshman praised a sculpture	2.76
Then an eagle surprised a kitten		Then a jury judged a gymnast	
Then a clergyman mocked a pessimist		Then a president calmed a baby	
Then a vampire thrilled a cardiologist		Then an enthusiast pleased a scholar	

*Average motion ratings obtained from Amazon Turk surveys are presented next to the stimuli*.

#### Word-comprehension stimuli

Words consisted of manner of motion verbs (“to bounce” *n* = 50), emission verbs (“to clang” *n* = 50), perception verbs (“to stare” *n* = 50), animal nouns (“the giraffe” *n* = 50), event nouns (“the hurricane” *n* = 50), and plant nouns (“the cactus” *n* = 50). See Table 1A for all word stimuli.

Among verbs, manner of motion verbs were rated significantly higher than emission [*t*_(21)_ = 4.95, *P* < 0.0001] and perception verbs [*t*_(21)_ = 4.27, *P* < 0.0001]. Emission and perception verbs did not differ from each other [*t*_(21)_ = 0.44, *P* = 0.66]. Among nouns, both animals and events had significantly higher visual motion ratings than the plant nouns [animals vs. plants, *t*_(21)_ = 4.86, *P* < 0.0001; events vs. plants, *t*_(21)_ = 5.63, *P* < 0.0001]. Animals and event nouns did not differ from each other [*t*_(21)_ = 0.75, *P* = 0.46].

Based on the ratings, words were grouped into high motion verbs (manner of motion verbs), low motion verbs (perception and emission verbs), high motion nouns (animals and events), and low motion nouns (plants). Average visual motion survey ratings for high and low motion verbs and nouns are presented in Figure 1A. According to information obtained using the Celex database (Baayen et al., 1995), high and low motion words did not differ in frequency [*t*_(149)_ = 1.27, *P* = 0.21 nouns, *t*_(149)_ = 0.34, *P* = 0.73 verbs] or number of syllables [*t*_(149)_ = 1.73, *P* = 0.09 nouns, *t*_(149)_ = 1.49, *P* = 0.14 verbs]. Syllable lengths, frequencies, and visual motion ratings by category are listed in Table 2.

**Table 2:** **Behavioral data from the word comprehension experiment and the sentence comprehension experiment**.

	**Number of syllables**	**Frequency**	**Visual motion ratings**	**Similarity/plausibility ratings**	**Reaction time (ms)**
High motion verbs	1.34 (0.52)	1.03 (0.60)	4.00 (0.69)	2.08 (0.97)	1676 (431)
Low motion verbs	1.52 (0.77)	1.00 (0.52)	3.01 (1.25)	2.16 (1.03)	1722 (445)
High motion nouns	2.42 (0.79)	0.68 (0.58)	3.67 (1.10)	2.22 (1.00)	1650 (432)
Low motion nouns	2.16 (1.00)	0.55 (0.57)	2.50 (1.39)	2.20 (0.92)	1651 (428)
Low motion passages	n/a	n/a	4.85 (0.95)	1.36 (0.17)	1785 (250)
High motion passages	n/a	n/a	2.65 (1.38)	1.29 (0.13)	1831 (248)

*Means and standard deviations of behavioral data of all conceptual categories. Standard deviations are presented in parentheses next to the mean*.

### Tasks

#### Passage-comprehension task

Participants read passages consisting of four sentences and rated each sentence in the passage on semantic plausibility. Passages were presented visually, one sentence at a time. Participants rated each sentence as either 1 plausible, 2 possible but unlikely, or 3 impossible. Eighty of the sentences were intentionally constructed to be semantically impossible (e.g., “An optimist snipped a sky”). Impossible sentences were equally likely to occur in either high motion or low motion passages, with only one sentence per passage being impossible. Participants were told before the scan that some sentences would be obviously impossible, but that there were no correct answers. Prior to the scan each participant practiced by rating 64 sentences from 16 passages that were not included in the actual fMRI experiment (32 high motion sentences and 32 low motion sentences). Participants indicated their responses on an MRI-compatible button pad. Each passage constituted one 12-s block of the task with each sentences presented for 3 s. Passages were separated by 10 s of fixation. The task was completed in six 6-min and 2-s runs with 20 passages per run. Blocks alternated between the high motion and low motion conditions and condition order was counterbalanced across all runs and across participants. Block order was randomized across participants.

#### Word-comprehension task

Participants next completed 3–4 runs of a word comprehension task. To ensure that participants were attending to the meanings of the words, they performed a meaning similarity judgment task during the MRI scan. Participants heard pairs of words and rated them on similarity in meaning on a scale of 1–4 (1 being very similar and 4 being very dissimilar). Each of the 300 words was presented twice during the experiment, each time paired with a different word. During each run 14 blocks were presented, and each block consisted of five word pairs of one word-type (animals, plants, manner of motion verbs, etc.). Each block was 18 s long (with each word pair presented for 3.6 s) and blocks were separated by 14 s of fixation. The order of the blocks was counterbalanced across participants. Participants also heard pairs of non-meaningful sounds (backwards speech) and judged sound similarity. Non-meaningful sounds were added as a baseline for a separate study and were not included in the analyses of the present paper. Words and sounds were presented over headphones and participants responded by pushing keys on an MRI-compatible button pad.

#### Visual motion localizer

After the sentence and word-comprehension tasks, participants saw 1–2 runs of visual motion localizer. Participants performed a 1-back task while viewing point-light animations in three conditions. The biological motion condition consisted of point-light walkers: light-points marked joint positions and their motion resembled human actions such as (walking, running, or jumping rope). For the random motion condition, the same points of light began motion from scrambled positions leading to a percept that did not resemble human actions (Grossman et al., 2000). In a third non-motion condition, the points remained in the same position on the screen and changed in luminance by fading in and out. The animations were blocked by condition; each block lasted 12 s (1.5 s per animation) separated by 12 s of fixation. Each run lasted 7 min and 24 s.

#### Functional magnetic resonance imaging data acquisition and analysis

Structural and functional data were collected on a 3 Tesla Siemens scanner at the Athinoula A. Martinos Imaging Center at the McGovern Institute for Brain Research at the Massachusetts Institute of Technology. T1-weighted structural images were collected in 128 axial slices with 1.33 mm isotropic voxels [repetition time (TR) = 2 ms; echo time (TE) = 3.39 ms]. Functional, blood oxygenation level-dependent (BOLD) data were acquired in 3 by 3 by 4 mm voxels (*TR* = 2 s; *TE* = 30 ms) in 30 near-axial slices. The first 4 s of each run were excluded to allow for steady-state magnetization.

Data analysis was performed using SPM8 and in-house software. The data were realigned, smoothed with a 5 mm smoothing kernel, and normalized to a standard template in Montreal Neurological Institute space. The modified-linear model was used to analyze BOLD activity of each subject as a function of condition. Covariates of interest were convolved with a standard hemodynamic response function (HRF). Nuisance covariates included run effects, an intercept term, and global signal. Time-series data were subjected to a high-pass filter (0.008 Hz).

BOLD signal differences between conditions were evaluated through second level, random-effects analysis. In whole-brain analyses, the false positive rate was controlled at *P* < 0.05 (corrected) by performing Monte Carlo permutation tests on the data (using a cluster size threshold with a primary threshold of 3; Nichols and Holmes, 2002; Hayasaka and Nichols, 2004).

Orthogonal functional ROIs were identified in individual subjects. Search spaces were defined based on the whole-brain group results, and individual ROIs were defined by taking all active voxels within a sphere around individual peaks in the search space. From the visual motion perception task, basic visual motion perception ROIs were defined based on the random motion > luminance change contrasts (bilateral MT/MST, IPL, and SPL). Bilateral biological motion ROI were also defined based on the biological motion > random motion contrast in the left and right STS (Grossman et al., 2000). With the exception of the LSTS, contrasts were thresholded in individual subjects at *p* = 0.001, *k* = 10 for the purposes of defining ROIs. If no voxels were observed at this threshold, the subject was excluded from the analysis. For left STS ROIs, contrasts were thresholded at *p* = 0.01, *k* = 10 because no ROIs could be defined at the higher threshold in most participants. This procedure resulted in the following number of subjects per ROI: LMT 15, RMT 15, LIPL 12, RIPL 13, LSPL 12, RSPL 13, RSTS 14, and LSTS 14.

We also defined an action-verb selective ROI along the pLMTG. As in prior work the pLMTG was defined based on the motion verbs > animals contrast from the word-comprehension task (Martin et al., 1995; Kable et al., 2002, 2005; Bedny et al., 2008). This resulted in 16 subjects with the ROI.

Region-of-interest (ROI) analyses were performed on the average of percentage signal change (PSC) relative to a resting baseline (for examples of similar analyses, see Saxe et al., 2006; Baker et al., 2007). For the data from the word comprehension task, we examined the PSC from TR 6 through 18; for the sentence comprehension task, the PSC was averaged from TR 4 to 16; for the visual motion perception task, PSC was averaged from TR 8 to 18. Tests carried out in the ROI analyses were not corrected for multiple comparisons.

## Results

### Behavioral results

#### Passage comprehension

Participants responded faster to the sentences in the high motion passages than to the sentences in the low motion passages [high motion *M* = 1.79 s, *SD* = 0.25 s, low motion passages *M* = 1.83 s, *SD* = 0.25 s; *t*_(16)_ = 3.23, *P* = 0.005]. High motion sentences were also rated as less plausible [high motion *M* = 1.36, *SD* = 0.17, low motion *M* = 1.29, *SD* = 0.13; *t*_(16)_ = 3.85, *P* = 0.001; Table 2]. The sentences that were intentionally constructed to be “impossible” received an average rating of 2.63 (*SD* = 0.30; with a rating of 3 for an impossible sentence). The average for the impossible sentences was significantly higher than the other sentences, which received an average rating of 1.26 [*SD* = 0.15; *t*_(16)_ = 17.87, *P* = 0.0001], indicating that participants were attending to the meanings of the sentences.

#### Word comprehension

There was no difference in reaction time between the low motion nouns and the high motion nouns [*t*_(13)_ = 0.14, *P* = 0.90]. The low motion verbs took longer than the high motion verbs [*t*_(13)_ = 2.70, *P* = 0.02]. There were no differences in the average within-category similarity ratings for high vs. low motion nouns [*t*_(13)_ = 0.45, *P* = 0.67] or high vs. low motion verbs [*t*_(13)_ = 1.41, *P* = 0.18; Table 2].

#### Visual motion localizer

Participants correctly detected repeating animations in 87% (*SD* = 18%) of the biological motion animations, in 86% (*SD* = 18%) of the random motion animations, and in 89% (*SD* = 19%) of static luminance change animations. Participants were faster at responding to static luminance (*M* = 0.85 s, *SD* = 0.27 s) than responding to random motion [*M* = 1.08 s, *SD* = 0.30 s; *t*_(15)_ = 2.81, *P* = 0.03] or biological motion animations [*M* = 1.07 s, *SD* = 0.26 s; *t*_(15)_ = 3.19, *P* = 0.006]. There was no significant difference between the two motion conditions [*t*_(16)_ = 0.61, *P* = 0.55].

### fMRI results

#### Visual motion localizer

Based on the random motion > luminance contrast, we defined the following ROIs in individual subjects (with average peak voxels): left IPL [−48 −40 21], right IPL [56 −38 23], left SPL [−29 −49 62], right SPL [30 −44 54], left MT/MST [−49 −72 4], and right MT/MST [49 −67 5]. Based on the biological motion > random motion contrast, we defined a right and left STS ROI in individual subjects, with average peaks voxels [−52 −62 6] and [57 −47 10], respectively.

**Figure 2:**
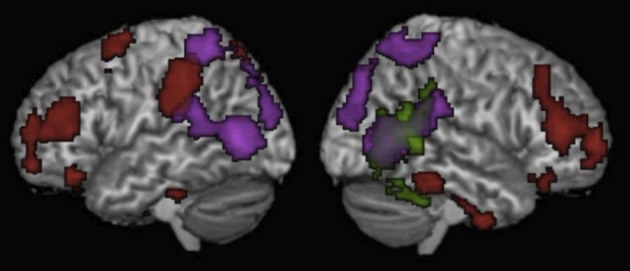
**Results of the whole brain analyses for Motion passages > Non-motion passages (Red), Biological motion > Static luminance (Green), and Random motion > Static luminance (Purple).** Results are thresholded at *p* < 0.05 (corrected for multiple comparisons) and displayed on a normalized template brain.

A whole-brain analysis of the random motion > luminance contrast revealed activity in traditional visual motion areas including bilateral MT/MST, bilateral SPL along the IPS and bilateral IPL (Table 3, Figure 2). The biological motion > random motion contrast revealed activity in the posterior aspect the RSTS (Table 3, Figure 2). At a lower threshold of *P* < 0.1 (corrected for multiple comparisons), activity was also observed in the posterior aspect of the left STS.

**Table 3:** **Results of whole-brain random effects analyses *P* < 0.05 (corrected)**.

**Contrast group**	***k***	***w***	***P*_combo_**	**Voxel peak *t***	***x***	***y***	***z***	**Brain area (Brodmann area)**
**BIOLOGICAL MOTION > RANDOM MOTION**								
	2308	8.82	0.0008	9.81	56	−50	10	Right superior temporal gyrus (22)
				7.02	48	−62	6	Right middle temporal gyrus (39)
				6.51	62	−46	2	Right middle temporal gyrus (22)
**RANDOM MOTION > STATIC LUMINANCE**								
	2202	8.82	0.0006	7.46	48	−64	4	Right middle temporal gyrus (37)
				5.83	46	−56	10	Right superior temporal gyrus (39)
				5.79	58	−46	14	Right superior temporal gyrus (22)
	1714	7.12	0.0032	7.16	−42	−64	10	Left middle temporal gyrus (19/37)
				6.80	−50	−70	4	Left middle occipital gyrus (19)
				5.26	−44	−40	24	Left inferior parietal lobule (13)
	3670	7.32	0.0026	6.36	−30	−52	60	Left superior parietal lobule (7)
				5.79	−22	−86	20	Left cuneus (18)
				5.73	26	−78	38	Right precuneus (19/7)
**HIGH MOTION PASSAGES > LOW MOTION PASSAGES**								
	361	8.82	0.0006	7.82	0	2	38	Cingulate gyrus
	284	8.82	0.0006	7.80	−26	32	−16	Left middle frontal gyrus (11)
	785	7.44	0.0028	6.81	4	−40	44	Right cingulate gyrus (31)
				6.38	−4	−32	46	Left paracentral lobule (31)
				3.46	4	−20	48	Right paracentral lobule (31)
	258	7.03	0.0036	6.74	24	30	−18	Right middle frontal gyrus (11)
				3.30	40	32	−20	Right inferior frontal gyrus (47)
				3.05	38	22	−20	Right inferior frontal gyrus (47)
	977	7.03	0.0036	6.41	28	64	2	Right superior frontal gyrus (10)
				6.38	50	44	12	Right middle frontal gyrus
				4.22	36	34	44	Right middle frontal gyrus (8/9)
	317	5.63	0.0148	6.17	50	−34	−22	Right inferior temporal gyrus (20)
				3.43	62	−40	−16	Right middle temporal gyrus (21)
	773	6.15	0.0086	5.98	−60	−36	46	Left supramarginal gyrus (40)
				4.88	−66	−26	26	Left supramarginal gyrus (40)
				4.83	−64	−34	28	Left supramarginal gyrus (40)
	350	4.95	0.0274	5.64	−8	−66	62	Left precuneus (7)
	300	4.77	0.0322	5.63	−30	−40	−18	Left fusiform gyrus (20)
				5.26	−30	−34	−26	Left fusiform gyrus (20)
**HIGH MOTION NOUNS > LOW MOTION NOUNS**								
	378	6.95	0.0042	6.59	52	−62	32	Right angular gyrus (39)
				5.10	46	−64	26	Right middle temporal gyrus (39)
	16	4.53	0.0424	5.71	−56	−16	−32	Left fusiform gyrus (20)
	549	5.06	0.0256	5.62	−46	−60	26	Left superior temporal gyrus (39)
				4.56	−52	−62	20	Left superior temporal gyrus (39)
**HIGH MOTION VERBS > LOW MOTION VERBS**								
	237	5.09	0.0244	5.98	−36	−86	32	Left superior occipital gyrus (19)
				4.14	−50	−74	14	Left angular gyrus (19/39)
				3.79	−44	−82	24	Left superior occipital gyrus (19)

#### Do visual motion brain regions respond to motion words?

We first asked whether visual motion areas (bilateral MT/MST, IPL, SPL, and STS) are sensitive to motion features of words. High motion nouns were compared to low motion nouns and high motion verbs to low motion verbs. (Verbs and nouns were compared separately because previous work has shown higher responses to verbs than nouns in nearby regions of the temporal and parietal cortex.) None of the visual motion ROIs in either temporal or parietal cortices showed significantly higher activity for high-motion verbs than low-motion verbs, or for high motion nouns than low motion nouns on average over the entire block (*t*'s < 2, *P*'*s* > 0.05; See Figure 1B, Table 4 for details).

**Table 4:** **Differences between high and low motion nouns and verbs in the visual motion regions of interest (not corrected for multiple comparisons)**.

	**High > Low motion nouns**	**High > Low motion verbs**	**High > Low motion passages**
LIPL	*t*_(11)_ = −0.38, *P* = 0.71	*t*_(11)_ = 0.21, *P* = 0.84	*t*_(11)_ = 0.02, *P* = 0.98
RIPL	*t*_(12)_ = 0.24, *P* = 0.81	*t*_(12)_ = 1.80, *P* = 0.10	*t*_(11)_ = 0.001, *P* = 0.99
LSPL	*t*_(11)_ = 1.23, *P* = 0.25	*t*_(11)_ = −0.21, *P* = 0.83	*t*_(11)_ = 1.66, *P* = 0.12
RSPL	*t*_(12)_ = 1.04, *P* = 0.32	*t*_(12)_ = 0.94, *P* = 0.37	*t*_(11)_ = 2.43, *P* = 0.03^*^
LMT	*t*_(14)_ = 0.15, *P* = 0.88	*t*_(14)_ = 0.17, *P* = 0.86	*t*_(13)_ = 0.95, *P* = 0.36
RMT	*t*_(14)_ = −0.30, *P* = 0.77	*t*_(14)_ = 1.84, *P* = 0.09	*t*_(13)_ = 0.31, *P* = 0.76
LSTS	*t*_(13)_ = 1.33, *P* = 0.21	*t*_(13)_ = −0.35, *P* = 0.73	*t*_(12)_ = −0.38, *P* = 0.71
RSTS	*t*_(13)_ = −1.35, *P* = 0.20	*t*_(13)_ = 0.17, *P* = 0.87	*t*_(13)_ = 1.13, *P* = 0.28

*Positive t-values indicate the mean for the high motion words was greater than that of the low motion words. Asterisks indicate a significant difference between the high and low motion conditions (P < 0.05)*.

In whole-brain analysis, high motion verbs lead to higher BOLD response than low motion verbs in the left superior occipital gyrus (−36, −86, 32, BA 19) and the posterior aspect of the left angular gyrus (−50, −74, 14, BA 39). High motion nouns compared to low motion nouns lead to higher response in the right middle temporal gyrus (46, −64, 26, BA 39), the right angular gyrus (52, −62, 32, BA 39), the left fusiform gyrus (−46, −60, 26, BA 20), and the left superior temporal gyrus (−52, −62, 20, BA 39). High motion noun and high motion verb responses did not overlap with each other, or with random motion or the biological motion contrasts from the visual motion localizer.

#### Do visual motion brain regions respond to motion passages?

The right SPL showed a small, but reliable increase in activity for high motion passages (*PSC* = −0.15) as compared to low motion passages [*PSC* = −0.17; *t*_(11)_ = 2.43, *P* = 0.03]. In the left SPL, the effect was in the same direction (higher for high motion sentences), but it was not significant [*t*_(11)_ = 1.66, *P* = 0.12].

There were no differences between the high and low motion passages in any of the other motion-responsive regions [right IPL *t*_(11)_ = 0.001, *P* = 0.99; left IPL *t*_(11)_ = 0.02, *P* = 0.98; right MT/MST *t*_(13)_ = 0.31, *P* = 0.76: left MT/MST *t*_(13)_ = 0.95, *P* = 0.36; right STS *t*_(13)_ = 1.13, *P* = 0.28: or left STS *t*_(12)_ = 0.38, *P* = 0.71] (see Figure 1B). Whole-brain analysis revealed higher signal for the high motion passages than the low motion passages in several regions in the temporal and parietal lobes (see Table 3 for full list of regions). However, none of these areas of activation overlapped with responses to random or biological motion, or with responses to high motion words (see Figure 2).

**Figure 3:**
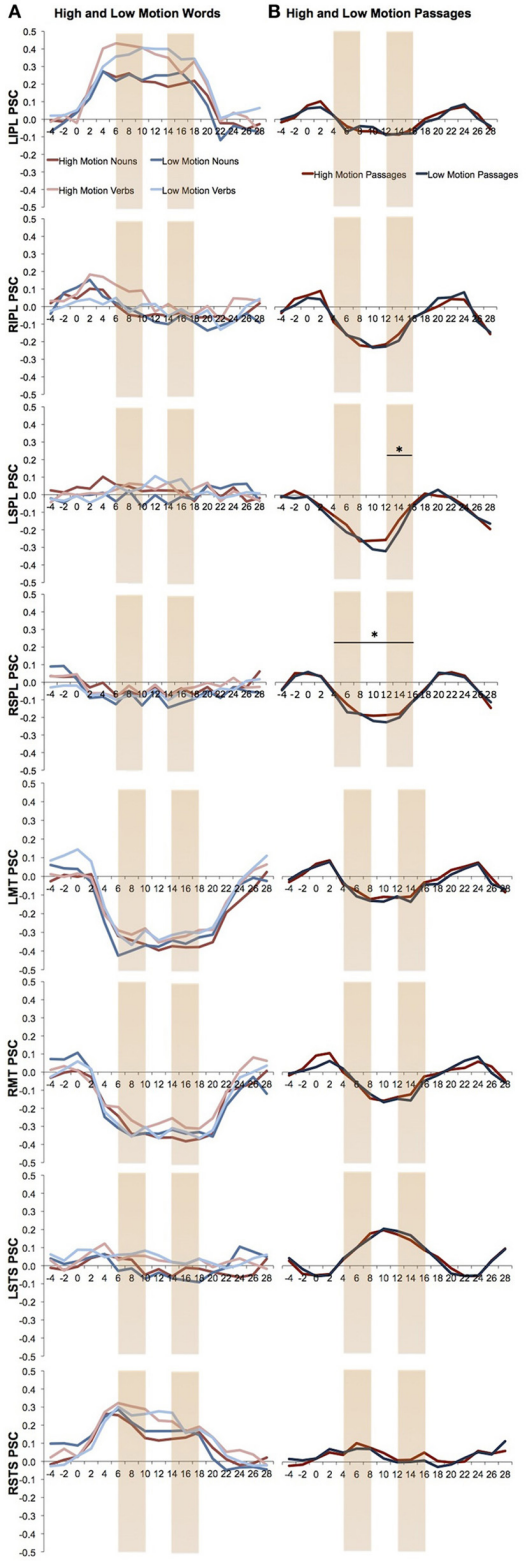
**Percent signal change over the time in the visual motion ROIs.** The first and last two TRs are marked. **(A)** Percent signal change of the high and low motion nouns and verbs. Analyses were averaged from TR 6 to18. **(B)** Percent signal change of the high and low motion passages. Analyses were averaged from TR 4 to16. Asterisks indicate a significant difference between the high and low motion conditions (*P* < 0.05).

One concern is that we might have missed possible responses to motion language by averaging activity over an entire block. Responses to high motion language could either attenuate over the duration of the block due to repetition suppression or increase due to build up. We therefore looked separately at responses to high and low motion language during the first and last two TRs of the block from each task. Note that the results of these analyses should be viewed as exploratory since there are a number of statistical comparisons and the analyses were not planned at the outset of the study. None of the visual motion ROIs showed a significant effect for both high > low motion nouns and high > low motion verbs in either the first two TRs or the last two TRs (Figure 3). Only the left SPL, which showed a trend in the block averaging analysis, also showed higher activity for the high motion than low motion sentences during the last two TR's of the block [*t*_(11)_ = 2.44, *P* = 0.03; Figure 3].

#### Does the pLMTG motion-verb area respond to perceptual visual motion?

**Figure 4:**
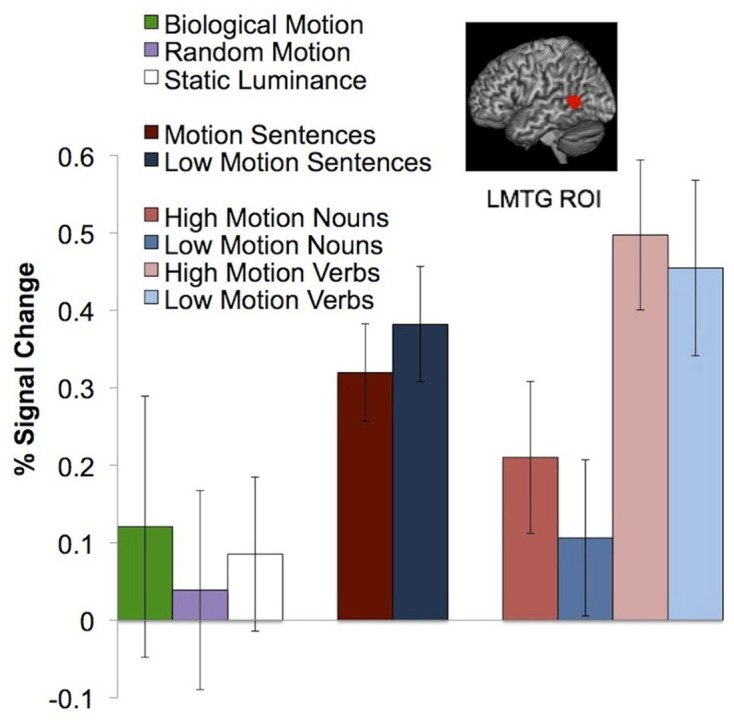
**Neural response in the pLMTG ROI to biological motion, random motion, static luminance, motion and non-motion passages, and high and low motion words**. Error bars represent a standard error of the mean.

As in previous studies, we observed a left middle temporal gyrus area that responds more to motion verbs than to object nouns (animals or plants; *P* < 0.05, corrected; Perani et al., 1999; Kable et al., 2002, 2005; Bedny et al., 2008). We defined the pLMTG ROI in individual subjects using the motion verbs > animals contrast, with average peak voxels [−52 −51 8]. The peak voxels from the whole brain analysis of this contrast were [−62 −52 8, BA22]. This pLMTG region did not respond to actual random [*t*_(12)_ = 0.24, *P* = 0.81, relative to static luminance] or biological motion [*t*_(12)_ = 0.15, *P* = 0.89, relative to random motion]. The pLMTG region also responded equally to high motion verbs and low motion verbs [*t*_(15)_ = 0.98, *P* = 0.33], high motion nouns and low motion nouns [*t*_(15)_ = −1.04, *P* = 0.32], and high motion passages and low motion passages [*t*_(14)_ = −1.56, *P* = 0.14; Figure 4].

## Discussion

The results of this study yield three main findings. First, we find that temporal lobe responses during comprehension of motion words and motion perception are distinct. Visual motion areas in the temporal cortex (i.e., MT/MST and the RSTS) show no response to single words with motion features or to passages that contain action verbs (see also Kable et al., 2002, 2005; Bedny et al., 2008). Conversely, a lateral temporal area that responds to action verbs (pLMTG) is insensitive to actual visual motion and does not distinguish between high and low motion words, or high and low motion passages. Second, parietal lobe areas engaged during visual motion perception are distinct from parietal regions that respond to motion words. Third, we find that passages are more effective than words at activating higher-order perceptual regions in the parietal lobe. Passages, but not words, activated the right SPL visual motion area. Since the plausibility task was easier for the high motion passages (reflected in faster reaction times), it seems unlikely that the SPL motion response reflects general processing difficulty or plausibility (despite the lower plausibility ratings for the motion passages). Notably the SPL response to language is small relative to neural responses to visual motion (*PSC* = 0.02), consistent with the possibility that it reflects spontaneous imagery. In sum, responses to linguistic depictions of motion are more likely for passages than for single words, and more likely in polymodal parietal areas than in modality-specific temporal areas. On the whole, however, neural responses to visually perceived motion and to linguistically described motion were largely distinct.

What do our findings reveal about the relationship of language and sensory perception? Consistent with many prior studies we find that language can influence activity in perceptual circuits (Meteyard et al., 2008; McCullough et al., 2012). Such observations argue against models of language and perception that assume modularity (Fodor, 1983). However, we also find that perceptual responses to language constitute a tiny fraction of the neural operations that are involved in language comprehension. Responses to language in the perceptual circuits are also distinct from responses during visual perception itself. Language effects are more pronounced in higher-order polymodal sensory areas than in early sensory areas. Even in these secondary perceptual regions, responses to language are weak relative to neural responses during vision.

By contrast to the current findings, some prior studies have reported responses to motion sentences in MT/MST and in the RSTS (Saygin et al., 2009; Deen and McCarthy, 2010; Humphreys et al., 2013). Why do some studies observe such effects while others (e.g., Wallentin et al., 2005) including the current study, fail to do so? We suggest that these differences may stem from the degree to which the stimuli promote spontaneous imagery. Just as imagery itself is a heterogeneous phenomenon (Kosslyn et al., 2001), so too perceptual responses during language processing vary depending on the details of the linguistic stimuli and task.

We hypothesize that linguistic stimuli that elicit specific and highly vivid visual images are required to activating early visual areas. In the present study neither the word nor the passage stimuli were likely to elicit such imagery. It seems unlikely that we did not pick the correct words to elicit high visual motion as our high motion verbs consisted of words describing manner of motion (ex: “to roll”). It is possible that words alone, out of context, are not enough to spontaneously elicit visual imagery even the passage stimuli in our study were not vivid enough to activate perceptual regions. The sequences of four sentences in the current study did not describe a single motion event, but rather a series of four different events (e.g., “The juror kicked a stool. Then a criminal jabbed a Dalmatian. Then a kid shut a door. Then a spaniel bounced a toy.”). By contrast, some previous studies used more extended, more coherent and more descriptive passages (e.g., Deen and McCarthy, 2010). Consistent with the possibility that long passages are better at eliciting imagery, a recent behavioral study found that effects of motion language on behavioral measures of motion perception increase with story length. Visual motion aftereffects grew as participants heard more sentences in a story, with no effects on perception for the first few sentences. Furthermore, effects of language on motion perception were higher for individuals who were better at generating vivid visual imagery (Dils and Boroditsky, 2010). Together, these results suggest that language is more likely to affect the visual system when the linguistic stimuli are sufficiently vivid to elicit spontaneous imagery.

Prior work also suggests that the emotional and motivational relevance of stimuli influences the likelihood of vivid mental imagery. One study found that MT/MST responds to sentences that describe motion toward the reader (e.g., “The car drives toward you”), but not to the same motion away from the reader (“The car drives away from you”; Rueschemeyer et al., 2010). Sentences describing motion toward the self also activated midline structures involved in motivational and emotional processing, suggesting that they had greater emotional salience. Descriptions of motion toward the self may encourage participants to anticipate possible visual motion events. For example, a sentence such as “Look out, a bicycle is heading right for you!” might prime visual motion and object perception circuits. Further research is necessary to test this claim.

There are also a number of ways in which task differences could influence whether linguistic stimuli activate visual motion regions. On the one hand, one might worry that some tasks could favor superficial encoding and thus artificially suppress activation of visual motion areas by language. We think that this explanation is unlikely, at least for the current tasks. Semantic similarity and plausibility judgments focus participants' attention on the meaning of the words and sentences. We have found that semantic similarity ratings of the kind collected here are highly systematic across participants, but are not well-explained by co-occurrence frequencies in corpus data (Koster-Hale et al., submitted). More generally, there is considerable evidence that word meanings are retrieved automatically, even when the task requires that participants ignore word meanings (e.g., the Stroop task; Stroop, 1935). Similarly, assessing sentence plausibility in the current task required not only retrieval of word meanings but integration of lexical and syntactic information to generate compositional meaning. Rather than being highly artificial, we suggest that the current tasks require deep semantic encoding and tap into processes typically involved in comprehension of words and sentences. Deep semantic encoding does not appear to be sufficient to activate perceptual circuits.

On the other hand, as with stimulus differences, some tasks may be more likely to activate perceptual areas because they are more likely to evoke vivid imagery. For example, spontaneous imagery might occur when linguistic information is relevant to visual perception, or when language elicits recall of specific episodic experiences, such as when hearing the sentence “Remember the way her dress swayed in the wind as she stood by the window?” We suggest that the extensive behavioral and neuroimaging literature on visual imagery is likely to provide a fruitful hypothesis space for studying interactions between language and perception (Kreiman et al., 2000; Kosslyn et al., 2001; Cui et al., 2007).

An interesting third possibility is that linguistic stimuli evoke responses in early visual motion areas only when participants are simultaneously engaged in perception of visual motion. Consistent with this idea, the two previous studies that observed responses to language in MT/MST involved simultaneously hearing motion language and seeing moving visual stimuli. Saygin and colleagues measured responses to motion sentences while participants were viewing videos of speakers (Saygin et al., 2009). Similarly, McCullough et al. reported responses in MT/MST to motion sentences while participants viewed videos of American Sign Language (McCullough et al., 2012). Parallel to these neuroimaging studies, a number of behavioral experiments have shown effects of language on visual perception in simultaneous visual and linguistic tasks (Meteyard et al., 2008). Together these findings suggest that linguistic descriptions of motion can modify ongoing MT/MST responses to visually perceived motion. A similar pattern has been observed with auditory motion: motion sounds by themselves do not drive responses to MT/MST, but they do modify MT/MST responses to visual motion (Sadaghiani et al., 2009).

In summary, the present data suggest that temporal and parietal responses to language and perception are largely non-overlapping. When language does evoke activity in perceptual areas, (1) rich linguistic stimuli such as passages are more likely to do so than single words and (2) effects are more likely to occur in higher-order polymodal areas than early visual areas.

### Implications for the relationship of perception and language

According to some versions of the embodiment hypothesis, concepts are solely comprised of perceptual schemas (Barsalou, 1999; Pulvermuller, 1999). For example, the concept of a phone consists of visual images of a phone shape and color, the memory of a phone sound as well as the tactile and motor memory of holding a phone (Allport, 1985). In this framework understanding words and sentences that describe motion depends on simulation of prior experiences of observing motion within the same modality-specific cortical systems that originally encoded the experience (Barsalou, 1999; Pulvermuller, 1999; Gallese and Lakoff, 2005; Speer et al., 2009). This view predicts that comprehension of motion words (e.g., “to skip”) and motion sentences (e.g., “The girl skipped down the hill.”) should necessarily be accompanied by activity in visual motion circuits (Barsalou, 1999; Pulvermuller, 1999). Contrary to this prediction, participants in our experiment made semantic similarity judgments about motion words and plausibility judgments about motion sentences without activating most visual motion areas. Moreover, a review of the literature suggests that responses to motion language in perceptual regions are small, variable, and clearly distinct from responses to actual visual motion. The neuroimaging evidence on the relationship of motion language and visual motion is thus inconsistent with a strong embodiment position.

Instead, neuroimaging findings are more consistent with the view that language and vision are distinct systems that interact during online processing. According to this account, language comprehension can occur independent of perceptual systems. Perceptual responses to linguistic stimuli reflect top-down effects of language on perception. This view makes several interesting predictions. First, within this framework it should be possible to observe effects of language on higher-order perceptual areas without effects in low-level perception areas, but not vice versa. This view also predicts that responses to language in perceptual circuits generally follow responses in language areas. For example, we expect that any response to language in visual motion areas will follow responses in the pLMTG. Impairment of processing in the pLMTG by brain damage or TMS should also impair downstream processing in perceptual areas, but not vice versa. Third, this view suggests that interactions between perception and language are not privileged. Rather they reflect the more general phenomenon whereby linguistic and non-linguistic information interact rapidly during online comprehension (e.g., Trueswell et al., 1994; McRae et al., 1998; Altmann, 1999).

In this paper we explore the relationship between language and perception by asking whether the same brain regions support these cognitive processes. What might be the limitations of such an approach? One possible objection is that neural and cognitive levels of analysis are entirely independent. On this view neural evidence cannot in principle speak to cognitive questions. A full discussion of this philosophical question is beyond the scope of the present article. We will merely point out that neuroscience has already provided considerable insights into the computations of the mind. Given the highly systematic relationship between neural function and cognition, it seems arbitrary to ignore biological evidence when considering issues of representation. A second version of this objection is particular to neuroimaging. The resolution of neuroimaging allows us to distinguish between neural areas and not between individual neurons. If the same area is discovered to support two different functions (e.g., syntax and semantics), it always remains possible that these functions would be separable at a higher level of resolution. However, when, as in the present case, despite the low spatial resolution of neuroimaging we find that two cognitive functions are supported by two different neural systems, it is not possible that they would appear to be supported by the same neural mechanism given higher spatial resolution. Despite these considerations it is important to point out that neuroimaging is only one kind of evidence for studying the relationship of language and perception. It is absolutely crucial to corroborate neuroimaging findings with complimentary techniques such as behavioral measures, brain stimulation, and temporally sensitive metrics (MEG, EEG).

### Conflict of interest statement

The authors declare that the research was conducted in the absence of any commercial or financial relationships that could be construed as a potential conflict of interest.

## Appendix

### Visual motion survey instructions

In this survey we're going to ask you what you think about when you hear or read stories. For each of the stories below we'd like to know to what extent you imagine visual motion in your mind's eye. Please rate each story on a scale of 1 to 7. If you could clearly visualize motion while reading the story, choose a number closer to 7. If you could not clearly visualize movement, choose a number closer to 1.

1 2 3 4 5 6 7

Low Medium High

There are no correct answers in this survey. We're interested in your opinion. Please give us your best estimate in your judgment and use the full range of the scale during the experiment (using all of the different numbers from 1 through 7).

To give you a sense of the task below, here are some examples:

Joe was playing soccer, he slid in to steal the ball; he kicked the ball away from the opposing player, got to his feet and began dribbling down the field.

Most people rate the story above as a 6.

Ellen took an important exam yesterday. She needed to pass in order to graduate. She passed and was very happy.

Most people rate the story above as a 1.

The list of stories appears below. There are a total of 96 stories. Thank you for completing this survey.
